# Supplementary material for: Evaluation of Probiotic Properties of Pediococcus acidilactici M76 Producing Functional Exopolysaccharides and Its Lactic Acid Fermentation of Black Raspberry Extract
Source: Microorganisms. 2021 Jun 23;9(7):1364. doi: 10.3390/microorganisms9071364 (PMC8304599; doi:10.3390/microorganisms9071364)
Supplement: Supplementary file 1 [file microorganisms-09-01364-s001.zip › microorganisms-1248035-supplementary.pdf]

Supplementary Materials

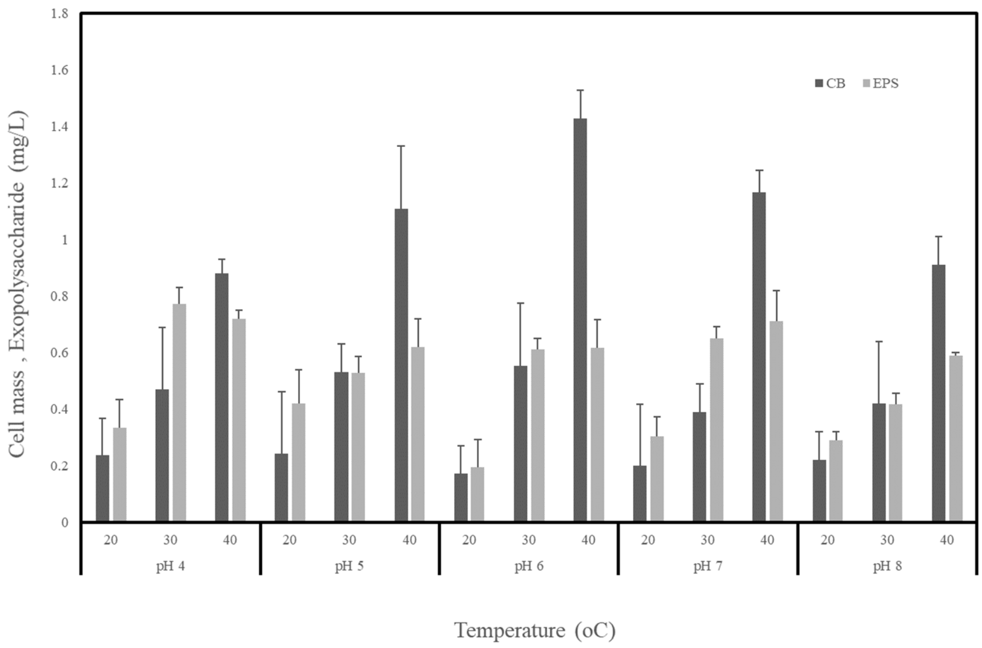

Figure S1. Cell mass and exopolysaccharide production of *Pediococcus acidilactici* M76 on at each pH and sugar concentration (°Brix).
